# Differential regulation of Dlg1, Scrib, and Lgl1 expression in a transgenic mouse model of ocular cancer

**Published:** 2008-12-19

**Authors:** V. Vieira, G. de la Houssaye, E. Lacassagne, J.L. Dufier, J.P. Jaïs, F. Beermann, M. Menasche, M. Abitbol

**Affiliations:** 1 Université Paris-Descartes, CERTO, Centre de Recherches Thérapeutiques en Ophtalmologie de la Faculté de Médecine Paris-Descartes-site Necker, EA n°2502 du Ministère de la Recherche, AP-HP, Paris, France; 2 Service d’Ophtalmologie, Hôpital Necker Enfants Malades, Paris, France; 3 Université Paris-Descartes, Faculté de Médecine Paris-Descartes, EA n°4067 du Ministère de la Recherche, AP-HP, Service de Biostastistiques et Bioinformatique du CHU Necker-Enfants-Malades, Paris, France; 4 Swiss Institute for Experimental Cancer Research (ISREC), Epalinges, Switzerland

## Abstract

**Purpose:**

Discs large (*dlg*), scribble (*scrib*), and lethal giant larvae (*lgl*) are major suppressor genes in *Drosophila melanogaster*. They encode proteins that regulate cell polarity and cell proliferation in *Drosophila* and mammals. However, their basic oncogenic roles have not yet been established in mouse epithelial ocular cancer. We evaluated the potential implication of these proteins in tumorigenesis of adenocarcinomas originating from the retinal pigmented epithelium of the Trp1/Tag transgenic mouse model. We examined the changes in the distribution and levels of these proteins in mouse ocular tissues from the Trp1/Tag mouse model.

**Methods:**

The expression patterns of theses genes and their corresponding proteins in normal mouse ocular tissues were studied by in situ hibridization and immunohistofluorescence experiments. In addition, variations in mRNA and proteins levels and protein distributions for Dlg1, Scrib, and Lgl1 were analyzed in the ocular tissues from Trp1/Tag transgenic mouse model by reverse transcription polymerase chain reaction (RT–PCR), western blot analysis, and immunohistofluorescence.

**Results:**

We found that mouse Dlg1, Scrib, and Lgl1 are widely distributed in normal ocular tissues, particularly in retinal neurons. We found that the three proteins are mislocalized in retinal layers during ocular carcinogenesis. These mislocalizations were correlated to the early dysplastic stages of ocular tumorigenesis. Additionally, the mislocalization of each protein was associated with its downregulation. Decreased levels of these proteins may be considered as late-stage markers of the disease but also as markers of the invasive stage of this cancerous process. This downregulation may be involved in epithelial*-*mesenchymal transition in this mouse ocular tumoral model. This would be consistent with the downregulation of E-cadherin and upregulation of N-cadherin expression observed in this model.

**Conclusions:**

This is the first study to demonstrate the involvement of *Dlg1*, *Scrib*, and *Lgl1* in a mouse with ocular adenocarcinoma and the simultaneous involvement of these proteins in the same cancer. Our results indicate that both the mislocalization and downregulation of these proteins may be involved together in ocular carcinogenesis.

## Introduction

Epithelial cells display an apico-basal polarity that is required for their correct epithelial structure and function [1]. Moreover, loss of epithelial polarity and architecture is one of the hallmarks of aggressive and invasive cancers. Polarity is mediated by the presence of different cell junctions and dependent on the formation of multiprotein complexes at the cell membrane. Most of our understanding about the link between cell polarity and the control of proliferation comes from studies on the *Drosophila melanogaster* genes, discs large (*dlg*), scribble (*scrib*), and lethal giant larvae (*lgl*), and their protein products [2,3]. Mutation analysis of these three genes in *Drosophila melanogaster* has revealed a link between the regulation of cell polarity and the control of cell proliferation. Indeed, mutations in any one of these genes cause loss of apico-basal cell polarity, loss of the columnar monolayered organization, and hyperproliferation, forming large neoplastic cell masses in the larval brain and imaginal epithelia [4-6]. These findings in *Drosophila* models have led to the classification of these three genes as tumor suppressor genes in *Drosophila* [2].

There are four closely related homologs of *Drosophila melanogaster* dlg in mammals (Dlg1–4), two distinct lgl homologs (Lgl1 and Lgl2), and only one scrib in higher vertebrates [7-11]. The Dlg, Scrib, and Lgl tumor suppressor proteins are conserved between species in terms of both sequence and function. Indeed, the mammalian homologs of these proteins can rescue their respective *Drosophila* mutants. Thus, the genetic pathways involved and their roles in the regulation of cell polarity and mammalian cell proliferation seem to be evolutionarily conserved [12-14]. Dlg1 and Scrib are mouse orthologs of discs large and Scrib in the *Drosophila*, respectively. Two Lgl homologs, Lgl1 and Lgl2, have been described in the mouse. Lgl1 is ubiquitous whereas Lgl2 has a tissue-specific distribution. Therefore, Lgl1 is considered to be the mouse ortholog of *Drosophila* lgl [15]. Dlg and Scrib are both PDZ (PSD-95/Dlg/ZO-1) proteins. PDZ proteins contain a common PDZ domain that is a protein recognition domain of approximately 80–90 amino acids [16]. Dlg belongs to the MAGUK (membrane-associated guanylate kinase) protein family. It is composed of several domains including three PDZ domains, a SH3 (Src homlogy domain 3) domain, a protein 4.1 binding motif, and a guanylate kinase (GUK) domain [17].

Scribble is a membrane-associated scaffolding protein belonging to the LAP (leucine-rich repeats and PDZ domain) protein family. It contains 16 LRRs (leucine-rich repeats) and four PDZ domains. In situ hybridization experiments on fetal mice have revealed that the mouse ortholog of *Drosophila* scrib, Scrb1, is expressed in several tissues including the thymus, testis, kidney, esophagus, stomach, and eye [18].

Lgl proteins contain several conserved functional domains including homo-oligomerization domains, a cluster of phosphorylation sites, and four WD42 repeats [19]. Lgl is localized at the basolateral membrane domain of *Drosophila* epithelial cells [2,20]. However, it is not yet known whether mammnalian Lgl interacts directly with either Scrib or Dlg. Although the functions of these proteins seem to be conserved in mammalian polarized cells (epithelial cells and neurons), it has been suggested that these proteins function cooperatively during the establishment of basolateral membrane identity and during apico-basal polarization.

Several lines of evidence demonstrate that the human proteins, hDlg, hScrib, and hLg1, play a role in human epithelial cancers and that decreased expression of the genes encoding these proteins is correlated with tumor progression [21-24]. To the best of our knowledge, although the roles of these three proteins have been thoroughly investigated in several cancers, a potential link among the possible dysregulation of these genes’ expressions, their corresponding protein distributions, and levels with the emergence, and the progression of ocular tumors has not been established. A transgenic mouse model has been produced, leading to the development of retinal pigmented epithelium (RPE) adenocarcinomas [25]. This model was generated by inserting a 1.4 kb fragment of Trp1 (tyrosinase-related protein 1) promoter fused to the large T antigen of SV40, which is a transforming sequence, into the Y chromosome. All transgenic mice were thus male. The Trp1 enzyme is expressed in melanocytes/melanoblasts and the retinal pigmented epithelium (RPE) during development. Expression of the large T SV40 antigen was first detected at embryonic stage (E) 10.5, and the first RPE abnormalities were observed at E15.5. Several preneoplasic foci are observed at this stage in the RPE. Proliferation of tumor cells occurs in the optic nerve region and near the anterior chamber where they expand into a large mass of neoplasic tissue. The tumor consists of epithelioid cells arranged in a tubular fashion resembling human RPE adenocarcinomas. At one month of age, transgenic mice exhibit visible enlargement of the eye, leading to the complete degeneration of the eye after two months of age. Tumor cells invade the optic nerve, and the mice die at three months of age. Metastatic invasion of the brain and lymph nodes are seen in all mice, and the invasion of the spleen occurs in about 30% of these mice [25]. The Trp1/Tag mouse model appears to be a very convenient model to study the involvement of Dlg1, Scrib, and Lgl1 proteins in ocular carcinogenesis including tumoral invasion.

Here, we investigated the deregulation of their mRNA and their corresponding protein levels as well as their localization in tumor-affected eyes by reverse transcription polymerase chain reaction (RT–PCR), western blot analysis, and immunohistochemistry. We tried to establish the contribution of each protein to tumor progression in the Trp1/Tag mouse model. We found that the three proteins were mislocalized in retinal layers during ocular carcinogenesis, which could be correlated to the early dysplastic stages of ocular tumorigenesis whereas levels of mRNA and protein levels for these proteins were reduced in tumoral eyes but only at later stages. Our study is the first to demonstrate the involvement of Dlg1, Scrib, and Lgl1 proteins in a mouse of ocular adenocarcinoma and the simultaneous involvement of these proteins in a same cancer.

## Methods

### Animals and tissues

All animals were handled in compliance with the Association for Research in Vision and Ophthalmology (ARVO) statement for use of animals in ophthalmic and vision research. CB6 and Trp1/Tag mice were kept at 21 °C with a 12 h light/dark cycle and fed ad libitum. We studied control CB6 mice and transgenic TRP1/Tag mice between the ages of postnatal stage (P) 15 and three months.

### RNA extraction and reverse transcription polymerase chain reaction

We collected one eye from five different mice each from the control and Trp1/Tag groups for each stage studied (P15, P20, P25, P30, and P90) and analyzed them independantly. Total RNA were extracted from these eyes using the TRIzol reagent (Invitrogen, Cergy-Pontoise, France) according to the manufacturer's recommendations. Five different samples were obtained for each control and Trp1/Tag group and for each stage. Total RNA (1 μg) was reverse-transcribed using an oligodT primer with SuperScript II RNase H reverse transcriptase (Invitrogen-Gibco) in a total reaction volume of 20 μl. cDNA products were amplified by PCR in a reaction volume of 10 μl containing 1 μM of each primer, 0.5 U Taq DNA polymerase (Invitrogen-Gibco), 10X PCR buffer, 1.5 mM MgCl_2_, and 0.25 mM dNTP (Promega, Charbonniéres-les-Bains, France).

Murine *Dlg1* specific primers (forward 5′-GAG CAT TGC ATC TGT TGG-3′ and reverse 5′-AGT GCA GCT GCT GCT TGTT −3′) were chosen to amplify a 1,557 bp fragment. Murine *Lgl1* was also amplified using the primers 5′-CAT CGC TTC CTG TGT CTT CA-3′ and 5′-AGG TTC CGC AGT TCT TCT CA-3′ to amplify a 500 bp fragment. In addition, *Scrib* primers (forward 5′-TGT CAG TGT CAT CCA GTT CG −3′ and reverse 5′-CCT CGT CAT CTC CTT TGT AG −3′) were designed so as to amplify an 897 bp fragment.

Murine *E-cadherin* (forward 5′-TAC TGC TGA GCT AAC CCA TG −3′ and reverse 5′-TTC ATC AGG ATT GGC AGG AC −3′) and *N-cadherin* (forward 5′-ACG GTG TAT GCT GTG AGA AG −3′ and reverse 5′-AGG CTT TGA TCC CTC TGG AA −3′) primers were also chosen to amplify specific 541 and 591 bp fragments, respectively.

*Cyclophilin A* and *GAPDH* were amplified as internal controls for comparative purposes. The *cyclophilin* primers (forward 5′-TGG TCA ACC CCA CCG TGT TCT TCG-3′ and reverse 5′-TCC AGC ATT TGC CAT GGA CAA GA-3′) were chosen to amplify a 311 bp fragment (Invitrogen-Gibco). *GAPDH* primers (forward 5′-GTT GCC ATC AAC GAC CCC TTC AT −3′ and reverse 5′-ATC CAC AGT CTT CTG GGT GGC A −3′) were also chosen. PCR products were analyzed by electrophoresis in 1% agarose gels and visualized by ethidium bromide staining under ultraviolet (UV) light.

Experiments were performed in triplicate for each eye. Band intensities were quantified with Image J software. The relative amount of mRNA was calculated for each eye as the ratio of the intensity of the each band to the *cyclophilin* band. The triplicates were then averaged.

At each stage, the mean of the five measurements of the control group was used as the reference, and individual ratios in the control and Trp1/Tag groups were relatively expressed to this value.

### Statistical analysis

All results are expressed as the mean±standard deviation (SD). The results were compared using analysis of variance (ANOVA) and Student’s *t*-test. A value of p<0.001 was considered statistically significant. All calculations were performed using the GraphPad Prism software (GraphPad Prism software Inc., San Diego, CA).

### Western blot analysis

Total protein was extracted from the eyes of adult CB6 and Trp1/Tag mice at P15, P20, P25, P30, and three months of age using extraction reagent (TRIzol; Invitrogen-Gibco) according to the manufacturer's instructions. Protein concentrations were determined with the Bradford protein assay. Protein (100 µg) from each sample was mixed with equal volumes of loading buffer (pH 6.8, 60 mM Tris, 10% glycerol, 2% sodium dodecyl sulfate, 5% 2-betamercaptoethanol, 0.01% bromophenol blue), boiled for 5 min, and then separated by electrophoresis in a 7% polyacrylamide gel containing SDS. Proteins were transferred onto a nitrocellulose membrane (Transblot Transfer Medium; Bio-Rad, Hercules, CA), which was blocked for non-specific binding by incubation with 5% skimmed milk for 1 h. Membranes were then incubated for 2 h with rabbit anti-SAP97 antibody (1:100; sc26532; Santa Cruz, Santa Cruz, CA), a goat anti-scribble antibody (1:100; sc11049; Santa Cruz), a rabbit anti-Lgl1 antibody (1/100; provided by Shigeo Ohno, Department of Molecular Biology, Yokohama, Japan), and a goat anti-β-actin antibody (C11; 1:1000; Santa Cruz). Membranes were washed in PBS-0.5% Tween-20 and incubated for 2 h with the appropriate horseradish peroxidase secondary antibody; anti-rabbit (sc2030, 1:5,000; Santa Cruz) or anti-goat secondary antibody (sc2033, 1:5,000; Santa Cruz). Proteins were then detected by chemiluminescence assay (ECL; PerkinElmer Life and Analytical Sciences, Inc., Courtaboeuf, France).

### Tissue preparation

Control CB6 and Trp1/Tag mice were killed by CO_2_ asphyxiation. Eyes were rapidly enucleated and fixed by incubation for at least 36 h in 4% paraformaldehyde (PFA) at 4 °C. They were embedded in paraffin and cut with a microtome (HM355; Microm, Les Ulis, France) into 5 μm sections, which were mounted on glass slides (Superfrostplus; Fisher Scientific, Illkirch, France), dried overnight at 37 °C, and stored at room temperature until use.

### Immunohistofluorescence

Tissue sections were deparaffinized in xylene and rehydrated through a graded series of ethanol. Sections were then incubated in a blocking solution overnight at 4 °C with a rabbit anti-Dlg1 (1:100; provided by J. Hell, University of Iowa, Iowa City, IA), a goat anti-Scrib (sc-11049, 1:100; Santa Cruz), or a rabbit anti-Lgl1 antibody (1;100, provided by Shigeo Ohno, Department of Molecular Biology, Yokohama, Japan). Sections were washed in 1X PBS and then incubated with the appropriate secondary antibody in a dark chamber as follows. For detection of SAP97 and Lgl1, sections were incubated for 1 h with 1:200 dilution of donkey anti-rabbit Alexa Fluor 488 (A21206; Invitrogen-Gibco). For detection of Scrib, sections were incubated with 1:200 dilution of donkey anti-goat Alexa Fluor 488 (A11055; Invitrogen-Gibco). Following incubation with secondary antibodies, sections were washed in the dark with 1X PBS and mounted in DAKOCytomation fluorescent mounting medium ((Dako, Glostrup, Denmark)). Sections were stored at 4 °C until viewing. The sections were viewed under a Leica SP5 confocal microscope (Leica, Rueil-Malmaison, France).

### In situ hybridization

Riboprobes were synthesized from *Dlg1*, *Lgl1*, and *Scrib* PCR fragments and labeled with a digoxygenin (DIG) RNA labeling kit 10X (Promega). In situ hybridization was performed on deparaffinized, rehydrated eye sections (5 µm) from control CB6 and Trp1/Tag animals. Sections were incubated overnight at 65 °C with the probes and washed with 1X Stringent Wash Concentrate (Dako) according to the manufacturer’s instructions. Sections were then incubated for 1 h at room temperature with a conjugate anti-DIG–AP (alkaline phosphatase) antibody and rinsed in PBS. Tissue sections were then incubated with the AP substrate, nitro-blue tetrazolium/5-bromo-4-chloro-3-indolyl-phosphate (NBT/BCIP), for 30 min in the dark. Slides were examined under a light microscope. We applied the same quantity of probe (sense and antisense) to each slide and treated all slides in a single experiment so that they could be compared. Experiments were performed in triplicate.

## Results

### Dlg1 mRNA and protein localizations in mouse retina

#### Localization of Dlg1 mRNA and protein in control mouse retina

**Figure 1:**
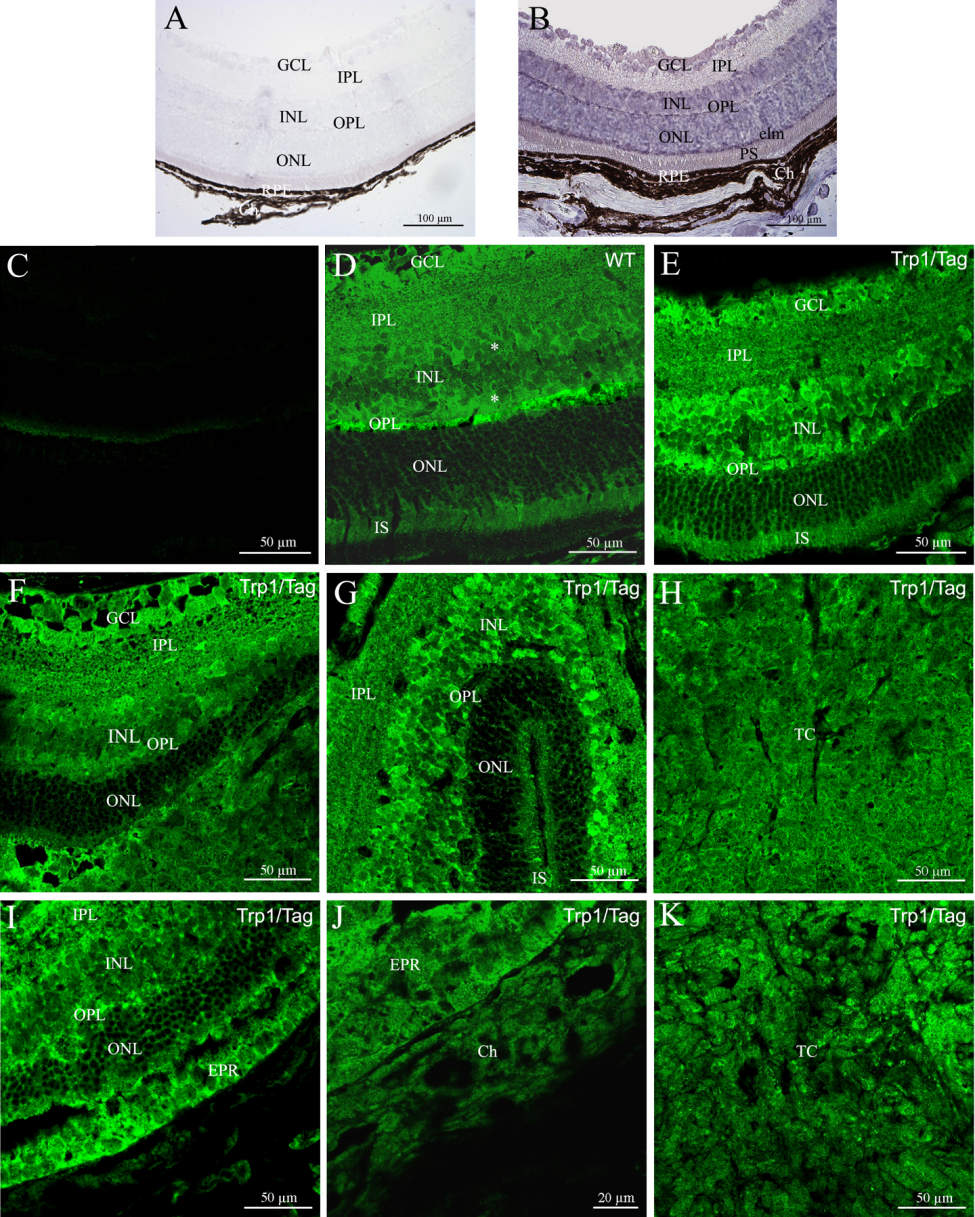
*Dlg1* mRNA distribution in the control retina and protein localizations in the adult retina and during development from control and Trp1/Tag mice using immunohistochemistry. **A** and **B** show *Dlg1* mRNA localization whereas **C**-**K** show the Dlg1 protein distribution in the adult mouse retina from the control mouse (**C** and **D**) and from the Trp1/Tag mouse model (**E**-**K**) at P15 (**E**), P20 and P25 (**F-H**), P30, and three months (**I-K**). **A** and **C** show the negative controls for **B** and **D**, respectively. Dlg1 mRNA and protein distributions were widely expressed in the ganglion cell layer (GCL), the inner nuclear layer (INL), the outer nuclar layer (ONL), and the photoreceptor inner segments (IS) of the control mouse retina. Very strong staining was detected in the outer plexiform layer (OPL) and the external limiting membrane (elm). However, faint staining of *Dlg1* mRNA was found in the inner plexiform layer whereas its protein was strongly detected. Futhermore, in the inner nuclear layer, *Dlg1* labeling was stronger at either side of the layer than in its center (indicated by an asterisk; **D**). Retina from the transgenic Trp1/Tag mouse model of ocular tumor continued to express Dlg1 protein (**E**-**K**). However, changes in the distribution of Dlg1 protein was observed from P20. In the central region adjacent to the mass of tumoral cells, Dlg1 immunolabeling in photoreceptor segments (IS) seemed to be reduced (**F**,**G**,**I**). A lower intensity of Dlg1 immunolabeling was shown in the OPL compared to the control retina (**F**,**G**,**I**). We also observed that immunolabeling in the INL appeared to be more diffuse in transgenics than in controls, possibly reflecting more labeling at the cortical regions than at membranous regions (**F**,**I**). In addition, from P30, the RPE and choroid (Ch) no longer stained positive for Dlg1 protein (**J**) in the posterior pole. The tumor cells (TC) had very faint staining for Dlg1 protein at these stages (**H**,**K**).

We studied the expression of murine *Dlg1* in whole eyes from control CB6 mice using in situ hybridization on longitudinally oriented adult mouse eye tissue sections. *Dlg1* mRNA was detected in the neuroretina (Figure 1A,B) but also in non-retinal ocular structures (data not shown). *Dlg1* mRNA was present within the neural retina, in the ganglion cell layer (GCL), the inner nuclear layer (INL), and the outer nuclear layer (ONL; Figure 1B). Very strong staining was also detected in the outer plexiform layer (OPL) and in the photoreceptor inner segments (IS). However, very faint *Dlg1* mRNA staining could be detected in the inner plexiform layer (Figure 1B). We detected *Dlg1* mRNA in the retinal pigment epithelium (RPE), choroidal melanocytes, and choroidal vascular endothelial cells (Ch; Figure 1B). The retinal Dlg1 immunostaining pattern throughout the neural retina mostly matched with the retinal *Dlg1* mRNA distribution detected by in situ hybridization with the exception of the inner plexiform layer (Figure 1D). Moreover, Dlg1 labeling in the inner nuclear layer was more intense at both sides of this layer than in its center (Figure 1D). The specificity of detection for mRNA and protein were confirmed by the negative control (Figure 1A,C).

#### Dlg1 protein localization in Trp1/Tag mouse eye

We explored the potential changes in the localization of Dlg1 protein in the retina of transgenic mice compared to the control mice (Figure 1E-K). At P15, Dlg1 protein distribution in the retina of transgenic mice was similar to that observed in the control mice of the same age (Figure 1E). At P20 and P25 (Figure 1F-H), the neuroretina of transgenic mice was highly disorganized at the posterior pole. This disorganization of the retina was associated with retinal dysplasia and the appearance of a tumor cell mass. Dlg1 labeling in the central retina remained unchanged compared to control mice whereas we observed a lower intensity of Dlg1 immunolabeling in the OPL than in control eyes (Figure 1F). Immunolabeling in the INL appeared to be more diffuse in the transgenic mice than in control mice, possibly reflecting a more intense labeling at cortical regions than at membranous regions. In the peripheral retina, retinal dysplasia was also associated with reduced Dlg1 labeling in the OPL as described in the central retina (Figure 1G). At these stages, tumor cells invading the neural retina from the transformed RPE stained positive for Dlg1 (Figure 1H). At P30 and three months of age (Figure 1I-K), this disorganization was amplified, spreading from the posterior to the anterior pole. However, at these stages, Dlg1 immunolabeling in the retina was less intense than in control mice at the same age (Figure 1I). In addition, Dlg1 immunolabeling appeared to be more diffuse in the INL (Figure 1I). Dlg1 immunolabeling was also reduced in the retina due to both the loss of photoreceptor outer and inner segments and the loss of a non negligible number of whole photoreceptor cells (Figure 1I). In the posterior pole, the RPE and choroid layers were still positive for Dlg1 as determined by immunohistochemistry (Figure 1J). The immunostaining of the tumor cell masses appeared to be less intense at these stages than at previous ones (Figure 1K). These observations were all validated in several sets of repeated experiments.

### Scrib mRNA and protein localization in mouse retina

#### Localization of Scrib mRNA and protein in control mouse retina

**Figure 2:**
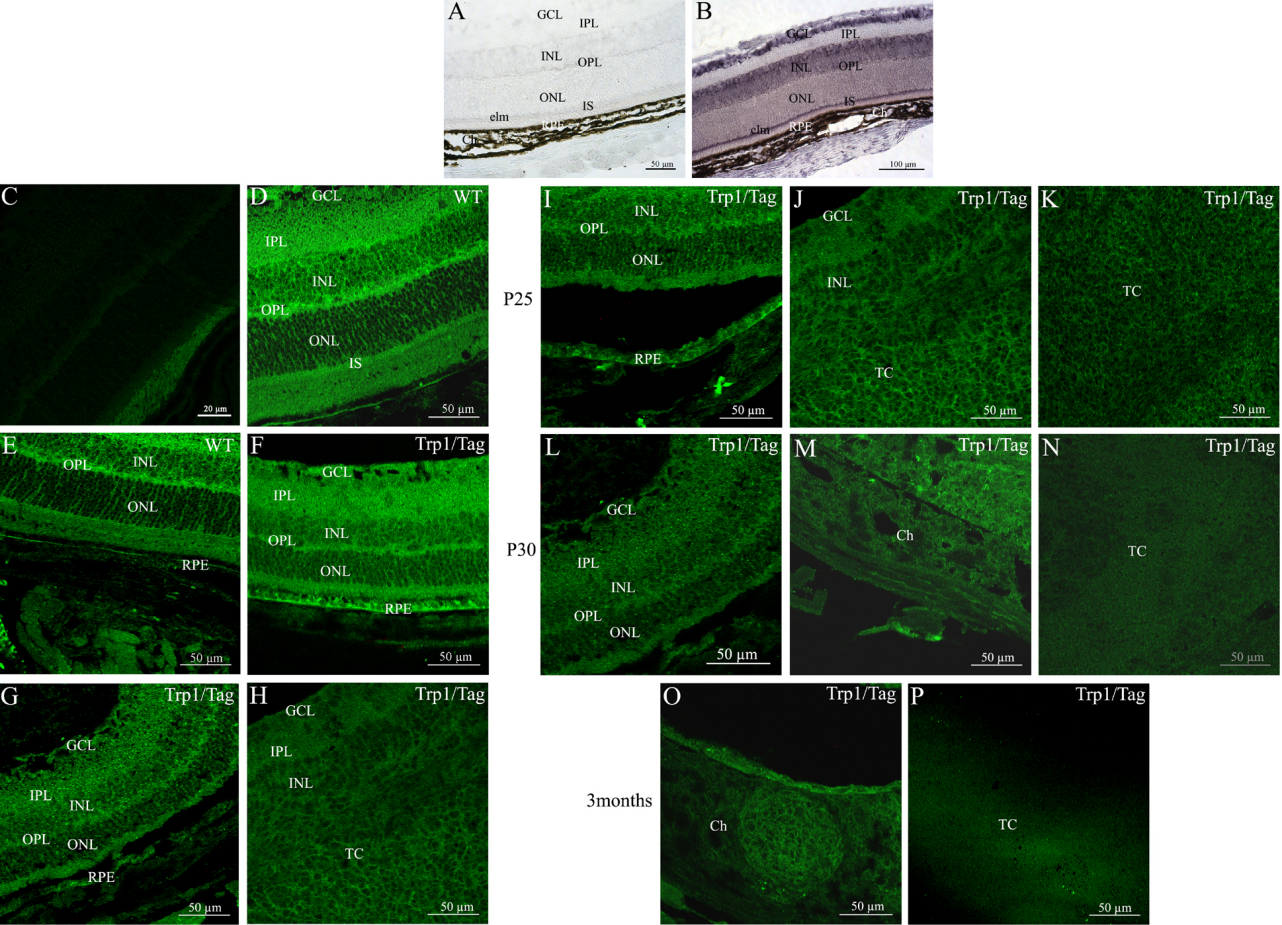
*Scrib* mRNA distribution in control retina and protein localizations in adult retina and during development from control and Trp1/Tag mice using immunohistochemistry. **A** and **B** show *Scrib* mRNA localization whereas **C**-**P** show Scrib protein distribution in adult mouse retina from the control mouse (**C**-**E**) and the Trp1/Tag mouse model (**F**-**P**) at P15 (**F**), P20 (**G**,**H**), P25 (**I-K**), P30 (**L-N**), and three months (**O**,**P**). **A** and **C** show the negative controls for **B** and **D**, respectively. In the retina, the *Scrib* mRNA and protein were observed in the ganglion cell layer (GCL), the inner nuclear layer (INL), the outer nuclear layer (ONL), and the outer plexiform layer (OPL). No mRNA expression was detected in the inner plexiform layer, but its protein was detected (in the IPL). Very strong levels of expression were detected in the photoreceptor inner segments (IS) and the external limiting membrane (elm). We also detected Scrib in the retinal pigment epithelium (RPE), choroidal melanocytes, and choroidal vascular cells (Ch). The neural retina continued staining positive for the Scrib protein in ocular tumor development in Trp1/Tag mice (**F**-**P**). However, from P15, strong staining was lost in the OPL (**F**,**G**,**I**,**L**). Scrib immunolabeling in the nuclear layers appeared to be more diffuse rather than restricted to cell membranes as observed in the control retina (**F**,**G**,**I**,**L**). The thickening of the pigmentary epithelium (RPE) was also positive for Scrib, which was predominantly localized to the cell membrane but also present in the cytoplasm (**F**). In addition, low levels of Scrib seemed to be present in the uvea (Ch; **M**). The intensity of the Scrib protein labeling in tumoral cells was markedly reduced and no longer restricted to cell membranes as described for P25. Scrib protein was detectable in tumor cells from P20 (**H**,**K**). However, at P30 and three months, no significant Scrib immunolabeling could be detected in tumoral cells (**N**,**P**).

*Scrib* mRNA distribution was also investigated in adult mouse retina. In the retina, we detected *Scrib* mRNA in the ganglion cell layer, the inner nuclear layer, the outer nuclear layer, and the outer plexiform layer. As described for *Dlg1* expression, very faint staining of *Scrib* mRNA could be detected in the inner plexiform layer (Figure 2B). However, *Scrib* was very strongly expressed in the photoreceptor inner segments (IS). We also detected *Scrib* mRNA in the retinal pigment epithelium, choroidal melanocytes, and choroidal vascular cells (Figure 2B). The specificity of *Scrib* mRNA detection was confirmed using the sense probe, which gave no significant hybridization signal in ocular structures (Figure 2A). The retinal Scrib immunostaining pattern throughout the neural retina mostly matched with its mRNA distribution detected by in situ hybridization, with the exception of the inner plexiform layer (Figure 2D,E). Additionally, a stronger level of immunostaining was found in the OPL and the photoreceptor inner segments/elm (external limiting membrane) structures (Figure 2D,E). No Scrib immunolabeling was observed in the negative control with the exception of the autofluorescent photoreceptor outer segments (OS; Figure 2C).

#### Distribution of Scrib protein in the Trp1/Tag mouse eye

We investigated potential differences in the pattern of Scrib protein in the retina during the development of the ocular tumors between Trp1/Tag transgenic mice and normal control mice at each stage (Figure 2F-P). At P15, the normal neural retina continued to display significant Scrib immunoreactivity (Figure 2F). However, intense Scrib immunostaining with a highly localized distribution pattern was observed in the OPL of normal adult mice whereas Scrib immunolabeling in the OPL of P15 transgenic mouse retinas was reduced. Similarly, Scrib immunolabeling in the retinal nuclear layers was restricted to the cell membrane region in control retinas but was more diffuse in transgenic mice (Figure 2F). We found intense Scrib immunostaining in the abnormally thickened RPE cells. Scrib immunoreactivity was particularly prominent nearby or within the cytoplasmic cell membrane in these cells but was also detected in the cytoplasm (Figure 2F). The same findings were observed for the P20, P25, and P30 retinas (Figure 2G,I,L). At the P20 and P25 stages, the tumor cells invading the neural retina exhibited significant Scrib immunostaining, which appeared to be localized to the cytoplasmic membranes (Figure 2H,J,K). The pattern of Scrib immunostaining remained unchanged at P30 in transgenic mice both in the neural retina and RPE compared to previous stages in Trp1/Tag mice (Figure 2L). However, at this stage, the uveal tract appeared to display very faint Scrib immunoreactivity (Figure 2M). Tumor cells still had detectable Scrib immunoreactivity, but a significantly lower level was observed than that observed at P25 (Figure 2N). Additionally, a diffuse pattern of Scrib immunoreactivity was observed in these cells. Unlike the preceding stages of tumor progression, no significant Scrib immunolabeling could be detected in tumoral cells at three months of age (Figure 2P). While the uveal tract displayed very faint Scrib immunostaining at P30, the tumor cell mass, which had clearly invaded this structure by this stage, displayed significant Scrib immunoreactivity (Figure 2O).

### Lgl1 mRNA and protein localization in mouse retina

#### Lgl1 mRNA and protein in control mouse retina

**Figure 3:**
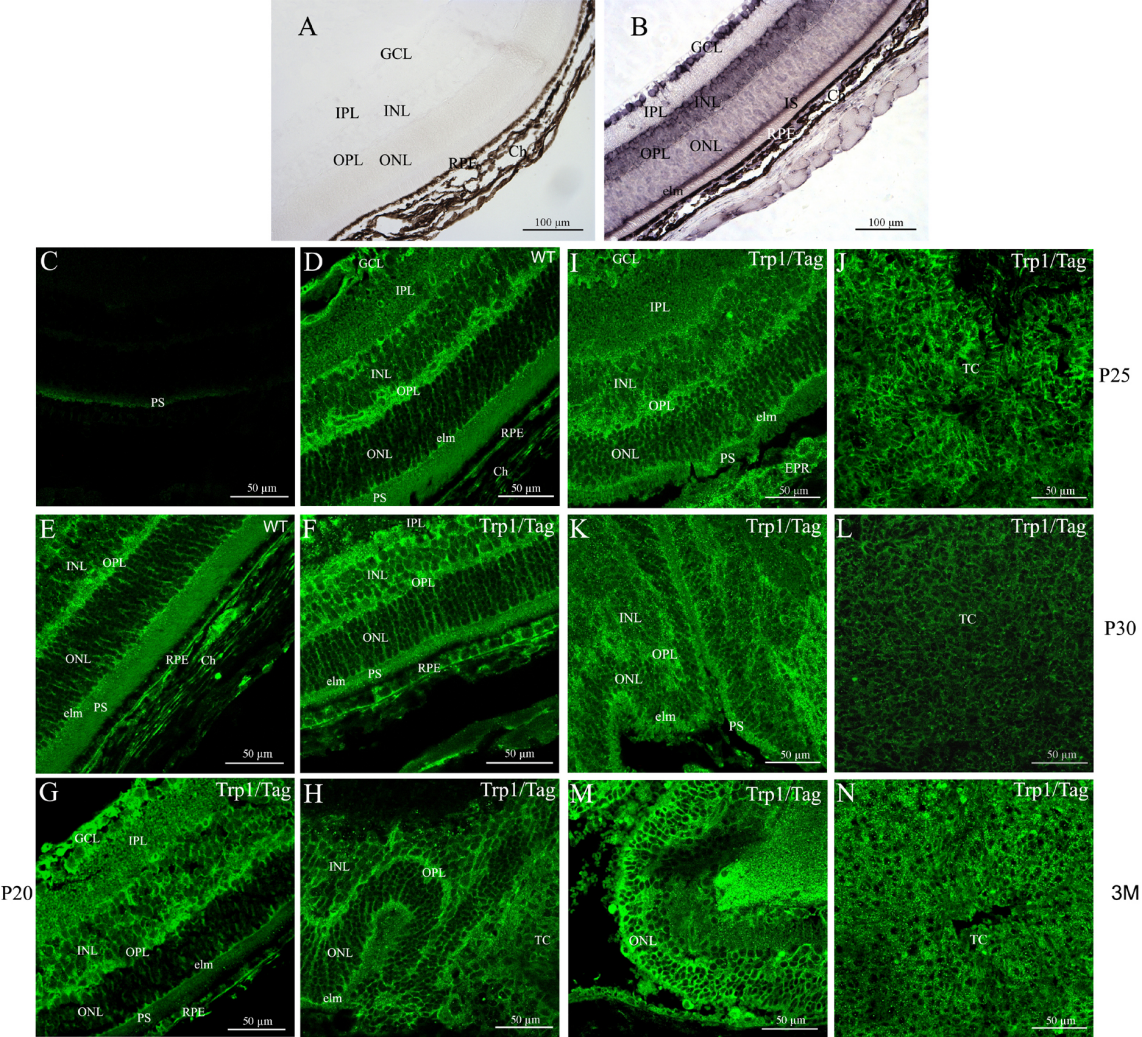
*Lgl1* mRNA distribution in control retina and protein localizations in adult retina and during development from control and Trp1/Tag mice using immunohistochemistry. **A** and **B** show *Lgl1* mRNA localization whereas **C**-**N** show Lgl1 protein distribution in the adult mouse retina from the control mouse (**C**-**E**) and Trp1/Tag mouse model (**F**-**N**) at P15 (**F**), P20 (**G**,**H**), P25 (**I**,**J**), P30 (**K**,**L**) and three months (**M**,**N**). **A** and **C** show the negative controls for **B** and **D**, respectively. In the retina, *Lgl1* mRNA and protein were found in the ganglion cell layer (GCL), the inner nuclear layer (INL), the outer nuclear layer (ONL), the photoreceptor inner segments (IS), the external limiting membrane (elm), the RPE, and the melanocytes and vascular cells in the choroid (Ch; **B**). No mRNA staining was found in the inner plexiform layer (IPL) or in the outer plexiform layer (OPL; **B**), but a weak trace of  protein was observed (**D**). Lgl1 labeling in the retinal nuclear layers seemed to be exclusively membranous (**D**,**E**). Lgl1 protein distribution in the retinas from the Trp1/Tag mouse model changed during tumoral development. In the INL and the RPE, Lgl1 immunolabeling appeared to be both membranous and cytoplasmic compared to the control retina (**F**,**G**,**I**,**K**). Lgl1 protein also appeared to be reduced in the INL, the OPL, and the elm from P25 (**I**,**K**). The tumor cells (TC) also displayed staining for Lgl1 protein during tumoral development in Trp1/Tag mice (**H**,**J**,**L**,**N**). However, very weak Lgl1 staining was detected in tumor cells at P30 (**L**).

Similar to for *Dlg1*, *Lgl1* mRNA was widely distributed in the adult retina (Figure 3). No *Lgl1* staining was observed in negative control using sense probe (Figure 3A). In the neural retina, *Lgl1* mRNA was found in the ganglion cell layer, the inner nuclear layer, the outer nuclear layer, and the photoreceptor inner segments (IS). Staining was stronger in the IS than in any other compartment of the neural retina (Figure 3B). We did not detect significant staining in the inner plexiform layer whereas weak mRNA levels were observed in the outer plexiform layer (Figure 3B). *Lgl1* mRNA staining was also observed in the RPE, the uveal melanocytes, and choroidal endothelial vascular cells (Figure 3B). The distribution of Lgl1 immunoreactivity throughout the normal adult mouse retina appeared to be very similar to that of its RNA distribution (Figure 3D). However, strong homogeneous Lgl1 immunostaining was observed in the outer plexiform layer and the photoreceptor inner segments. The external limiting membrane was also intensely immunostained for Lgl1 (Figure 3D,E). For the negative control, immunolabeling was only observed in the autofluorescent photoreceptor outer segments (Figure 3C).

#### Lgl1 protein localization in Trp1/Tag mouse eye

The distribution of Lgl protein was also determined in the Trp1/Tag retina tissue and compared to control one (Figure 3C-E). At P15, the cellular distribution of Lgl1 in transgenic mice appeared to be identical to that observed in the P15 normal control retinas (Figure 3F). However, in the thickening observed in the RPE, the staining seemed to be more closely linked to the cytoplasmic region than to the membrane of RPE cells (Figure 3F). The choroid had reduced levels of Lgl1 immunostaining at this stage. From P15, Lgl1 immunolabeling appeared to be more diffuse in the INL, possibly due to its presence both in the intracytoplasmic and membrane regions (Figure 3F,G,I,K). Additionally, Lgl1 immunolabeling of the INL and OPL appeared be less intense than in the control retinal tissue sections from P25 (Figure 3I,K).

Tumor cells had detectable Lgl1 immunoreactivity at P20, P25, P30, and three months of age (Figure 3J,L,N). However, at the P30 stage, the immunostaining of these tumor cell masses appeared to be less intense than at the others stages (Figure 3L).

### Downregulation of *Dlg1*, *Lgl1*, and *Scrib* expression during tumor development in Trp1/Tag transgenic mice

**Figure 4:**
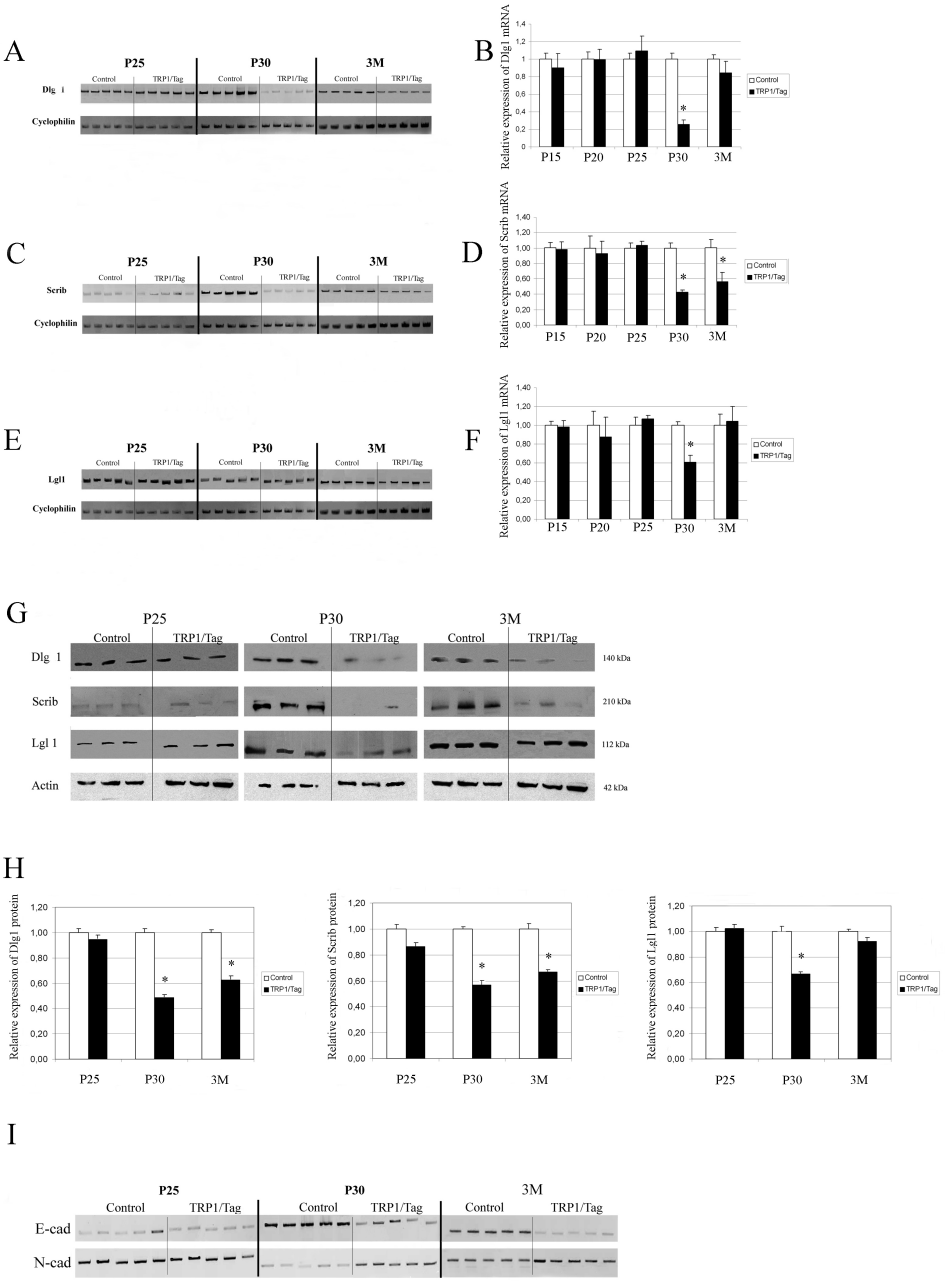
*Dlg1*, *Scrib*, and *Lgl1* mRNA and protein expressions in mouse whole eyes during tumor development and in control eyes. Semi-quantitative RT–PCR was used to determine the relative amounts of *Dlg1* (**A**), *Scrib* (**C**), and *Lgl1* (**E**) mRNA in mouse whole eye tissue at P25, P30, and three months in CB6 control and Trp1/Tag mice. *Cyclophilin* was used as an internal control. Relative levels were calculated as the ratio of the intensity of each PCR band to the *cyclophilin* band. **B**, **D**, and **F** shows means of the ratio of expression corresponding to **A**, **C**, and **E**, respectively. **G** shows corresponding western blot analysis. The blot was counterstained with anti-β-actin antibody as a loading control. Specific bands corresponding to Dlg1 (140 kDa), Scrib (210 kDa), Lgl1 (112 kDa), and β-actin (42 kDa) were detected in all extracts. **H** shows the relative levels of each protein, which were quantified and normalized using β-actin as the internal standard. **I** shows E-cadherin and N-cadherin expressions using RT–PCR in control and TRP1/Tag mice. Error bars indicate SEM. Asterisks indicate statistically significant results (p<0.001). Dlg1, Scrib, and Lgl1 protein levels were reduced in the whole eye from the adenocarcinoma model at P30 whereas only Dlg1 and Scrib protein were reduced at three months (**G**,**H**). E-cadherin expression in total RNA samples extracted from whole eyes was much lower in the Trp1/Tag model than in the control mice at the P30 and three month stages (**H**). An upregulation of N-cadherin expression was associated with E-cadherin downregulation at these stages (**I**).

Our findings from immunohistochemistry experiments suggested a decrease of Dlg1, Lgl1, and Scrib protein levels during the development of the ocular adenocarcinoma of the Trp1/Tag mouse model. Therefore, we determined the levels of the corresponding mRNAs in these mice using semi-quantitative methods. We used semi-quantitative RT–PCR to compare the amount of each mRNA in total RNA extracts from normal (CB6) eyes and tumor-derived eye tissue from Trp1/Tag transgenic mice at different stages (Figure 4). The levels of *Dlg1*, *Lgl1*, and *Scrib* mRNAs were analyzed at P15, P20, P25, P30, and three months of age. *Cyclophilin* was used as an internal standard for quantifying changes in levels for each class of mRNA (Figure 4). The relative amount of each mRNA was calculated as the ratio of the intensity of the each band to the *cyclophilin* band.

The PCR amplified product obtained using the *Dlg1*-specific primers produced a clear band of the predicted size, 1,557 bp. *Dlg1* mRNA was present at all postnatal stages studied in eyes from control and Trp1/Tag mice. We did not observe any changes in *Dlg1* expression at P15, P20 (data not shown), or P25 following normalization with respect to *cyclophilin* expression in Trp1/Tag mice compared to control ones at the same stages. However, *Dlg1* expression levels were four times lower in the Trp1/Tag model than in wild-type mice at P30 (Figure 4B). This reduction in *Dlg1* transcript levels was not observed at the age of three months in Trp1/Tag mice compared to the control mice (Figure 4A,B). The PCR-amplified product obtained using *Scrib*-specific primers produced a clear band of the predicted size, 897 bp. As described above for *Dlg1, Scrib* mRNA was present at all postnatal stages studied in control and Trp1/Tag mice (Figure 4C). The *Scrib/cyclophilin* mRNA ratio in whole eyes showed significantly lower levels of *Scrib* gene transcription in tumor-derived eye tissue from Trp1/Tag mice at P30 (2.3 times lower) than in normal control eyes at the same stage (Figure 4D). Notably, this decrease in *Scrib* gene expression was observed at the same stage of tumor development as that corresponding to the decrease observed in *Dlg1* mRNA levels. While the decrease in *Dlg1* transcription levels was only seen at the P30 stage, *Scrib* transcription levels were also decreased (1.9 fold) at the final three month stage compared to the control mice (Figure 4D). Similarly to *Dlg1* and *Scrib* expression, *Lgl1* mRNA was expressed at all postnatal stages studied in control and Trp1/Tag mice (Figure 4E). *Lgl1* transcripts levels in ocular total RNA samples extracted from Trp1/Tag model at P30 were significantly lower than in control samples at this stage. However, no significant decrease in *Lgl1* mRNA levels could be detected at the age of three months, unlike that observed for *Scrib* mRNA expression (Figure 4F). The same results were obtained using *GAPDH* as an additional internal control used for comparative purposes (data not shown).

### Differential decreases in Dlg1, Scrib, and Lgl1 protein levels during cancer progression in Trp1/Tag mice

To confirm our observations, we used western blot to determine protein levels (Figure 4G). Protein levels were analyzed in protein extracts from whole eyes of control and Trp1/Tag mice at the same stages (P15, P20, P25, P30, and three months) using the same antibodies used in immunohistochemistry experiments. No changes in protein levels were observed between P15 and P25 in Trp1/Tag mice compared to the control mice (data not shown). Consistent with the reduced mRNA levels at P30, only a low level of Dlg1 was detected in Trp1/Tag mice compared to the control mice at P30. However, whereas *Dlg1* mRNA was not reduced at three months of age in the ocular tumor total RNA samples, we found that the protein levels were still low at this stage compared to the control mice (Figure 4H). Similarly, Scrib protein levels were decreased at both the P30 and three month stages of tumor development in Trp1/Tag mice compared to control mice. Additionally, our data obtained for *Lgl1* mRNA expression were supported by western blot analysis. We found decreased Lgl1 protein levels in proteins extracts from tumor-derived tissue when compared to proteins samples from control mice at P30 only (Figure 4H).

In summary, both RT–PCR and western blotting revealed a downregulation of *Dlg1*, *Scrib*, and *Lgl1* in whole eyes from Trp1/Tag transgenic mice with an inverse correlation observed reproducibly between protein levels and the tumor invasion stage in our ocular tumor model.

### Downregulation of *E-cadherin* mRNA transcription

It has been long established that cancer development is associated with a loss of cell adhesion. In particular, a switch from E-cadherin to N-cadherin interactions contributes to a stroma-oriented cellular adhesion profile with increased tumor cell motility and invasive properties. Some studies suggest that Scrib and Lgl proteins can regulate *E-cadherin* expression in *Drosophila* [26,27]. We decided to analyze *E-cadherin* and *N-cadherin* expression by RT–PCR using specific primers. RT–PCR analysis showed that *E-cadherin* expression in total RNA samples extracted from whole eyes was much lower in the Trp1/Tag model than in control mice at the P30 and three month stages (Figure 4I). We also observed an upregulation of *N-cadherin* expression, which is associated with *E-cadherin* downregulation, at these stages (Figure 4I).

## Discussion

There is growing evidence to suggest that Dlg, Scrib, and Lgl proteins are important mediators controlling cell polarity and potential tumor suppressors in mammalian cells as described for their *Drosophila* homologs. A transgenic mouse model was previously developed in which the SV40 T antigen is produced under the control of the Trp1 promoter [25]. This model is characterized by tumor formation in the retinal pigment epithelium (RPE). The mice develop primary ocular tumors, which start to develop from the RPE before birth and progressively invade the optic nerve and metastasize in the brain, lymph nodes, and spleen. The Trp1/Tag model is relevant in carcinogenesis, mimicking the development of the equivalent human cancer (adenocarcinomas), and allowing the study of the dissemination process. The aim of our study was to investigate the gene expressions and protein distribution and levels for mouse Dlg1, Scrib, and Lgl1 during mouse tumorigenesis in this transgenic model of ocular cancer and to establish their potential involvement in the malignancy process. Specifically, we determined whether Dlg1, Scrib, or Lgl1 levels and distributions were altered during tumor progression.

We first found that Dlg1, Scrib, and Lgl1 are widely distributed in adult mouse retina. More specifically, these three proteins were detected in mouse retinal neurons. Furthermore, strong staining for Dlg1 and Scrib was observed in the same layers of the retina, the OPL, and photoreceptors inner segments (IS) from postnatal developmental stages to adulthood. These results complement those of Nguyen et al. [28] who found that Dlg1 and Scrib were colocalized in certain regions of the mouse retina. These findings suggest that Dlg1 and Scrib proteins act cooperatively. Importantly, we also detected strong staining for the Lgl1 protein in these same retinal layers. Our results seem to confirm that these three proteins may act altogether cooperatively in the same retinal layers. We demonstrated that each of the three proteins studied has a distinct distribution in the neural retina. Dlg1 was particularly abundant in the INL and OPL, and Scrib protein was predominant in the GCL and INL whereas staining for Lgl1 was strongest in the ONL. These different localization patterns suggest that these three proteins as well as cooperative functions in the regions where they are colocalized might also display distinct functions. These results also suggest that each of these proteins might be associated with distinct, specific subpopulations of neurons in the adult mouse retina. Further experiments using double immunolabeling experiments may help to confirm these hypotheses. Data collected from both *Drosophila* and mammals suggest that the role of the Dlg protein in clustering and trafficking receptors involved in neurotransmission have been strongly conserved in evolution [29,30]. Our observations in normal adult mouse retinas support the notion that Dlg1 may share the same functions of clustering and trafficking of synaptic proteins and may be required for normal retinal function. This is supported by previous studies showing that Dlg1 is essential for retinal response in *Drosophila* [31]. Mouse Scrib in retinal neurons may also retain some of the properties that they display in *Drosophila* in synaptic organization and plasticity [32]. Previous reports suggest that the Lgl protein may also have important functions in the retina related to its regulation of exocytosis through its interaction with target soluble N-ethylmalemide–sensitive factor attachment protein receptor (t-SNARE), a function that seems to be conserved between different species and cell types [33-35]. Indeed, mouse Lgl1 may act to restrict protein localization at particular membrane sites by regulating a major exocytosis pathway in retinal neurons. However, additional experiments will be required to determine whether all the potential functions of these proteins are related to synaptic functions.

We also examined these proteins in the ocular tissues of Trp1/Tag mice. We showed that immunoreactive staining for Dlg1, Lgl1, and Scrib in the retinal outer plexiform layer was lower in these mice than in controls. We also found reduced levels of Scrib and Lgl1 in the external limiting membrane. Consistent with these observations, we found reduced levels of the Dlg1, Scrib, and Lgl1 proteins in western blots at the P30 stage in Trp1/Tag mice. At this stage, tumor cells invade the optic nerve. Our results indicate that there is a correlation between decreased levels of these proteins and the invasion of adjacent tissues. These proteins display several crucial properties likely to be involved in major retinal neuronal functions such as synaptic formation, functioning, and plasticity. Thus, the effects of their downregulation should be assessed through examination of synaptic and spine structure morphology using specific immunohistochemical markers and combined with functional exploration of retinal functions based on electrophysiological analyses of Trp1/Tag mice.

Whereas the downregulation of Dlg1 and Lgl1 observed at P30 was no longer obvious at later stages in the progression of mouse ocular adenocarcinoma, the downregulation of Scrib mRNA and protein persisted. Consistent with our results obtained by semi-quantitative RT–PCR and western blots of total RNA and protein from whole eyes of transgenic mice, we observed a greater reduction in the intensity of the Scrib specific immunostaining in ocular tumor-derived tissue than for Dlg1 or Lgl1 in the same ocular tissue. The reasons for the differences observed in the regulation of these proteins remain unclear. Previous studies suggest that the roles of Lgl1 in controlling cell proliferation and apico-basal polarity in the developing eye of *Drosophila* are separable [36]. Indeed, the results exposed suggest that high levels of Lg1l are required to reduce proliferation whereas lower Lgl1 levels are needed for the maintenance of apico-basal cell polarity. Previous studies on Dlg1 mutants have also suggested that a specific threshold level of protein is required for cell polarity regulation whereas a higher level of protein is required for the control of cell proliferation [37,38]. However, the analysis of Scrib did not suggest that the two functions were independent. However, it should be noticed that the deletion of PDZ domains leads to only mild polarity defects and to moderate overproliferation [39]. The hypothesis proposed for explaining these facts is that Scrib acts primarily to regulate apico-basal polarity and that loss of polarity itself disrupts proliferation control. Thus, these two distinct processes seem to be coordinated and cannot be separated in *Drosophila* [39]. Further experiments will be required to determine whether this is the case in mammals.

In addition to the reduced protein levels observed in western blots, we showed that immunostaining for Dlg1, Scrib, and Lgl1 in the inner nuclear layer was more diffuse for Trp1/Tag retinas during tumor development than for wild-type mouse retinas. We also showed diffuse staining for Scrib in the outer nuclear layer. Dlg1, Lgl1, and Scrib proteins detected in the immunolabeled cells of the ONL and INL of control mouse retinas seemed to be normally localized close to the cytoplasmic membranes. Staining appeared to be more predominant in intracellular cytoplasmic regions in the ocular tumor mouse model. This abnormal mislocalization of Dlg1, Lgl1, and Scrib was also found in epithelial cells, the corneal epithelium, lens epithelium, and the RPE of the transgenic mouse model (data not shown). Only Dlg1 and Scrib proteins displayed an increased cytoplasmic distribution in tumor cells. Several previous studies have reported that Lgl1 protein follows this pattern of translocation in human malignant melanoma [27]. A similar process of translocation of the Dlg1 protein occurs in cervical intraepithelial neoplasia [23,40]. Additionally, the Scrib protein also changes its cellular location during the progression of colon cancer [21]. Our results support the importance of intracellular mislocalization of Dlg1, Scrib, and Lgl1 during cancer development as suggested previously [41,42]. The mechanism(s) contributing to these changes remain(s) to be determined. However, the potential involvement of atypical Protein Kinase C (aPKC) in a mechanism underlying Lgl1 mislocalization could be explored in our model. Under normal conditions, aPKC is localized at the apical domain and is able to phosphorylate Lgl1. This Lgl1 phosphorylation induces its detachment from the plasma membrane due to a conformational change. The displacement of phosphorylated Lgl1 results in the inhibition of Lgl1 function at the apical cellular domain and subsequently the inhibition of aPKC function by Lgl1 at the basolateral domain [43,44]. This regulation by aPKC was observed in epithelial cells [45] and mouse brain fibroblasts [46]. Additionally [42],  showed that the presence of aPKC in the cortex leads to the cytoplasmic release of Lgl1 and massive overgrowth in *Drosophila melanogaster*. In ovarian carcinomas, the aPKC ξ isoform displays an apical cortical distribution and Hugl1 is released from the membrane into the cytoplasm, reminiscent of events observed in the *Drosophila* model [42]. It appears likely that this mechanism also regulates Lgl1 intracellular localization in the mouse retina. This hypothesis could be tested in experiments determining whether the Lgl1 mislocalization observed in the tumor cells of our transgenic mouse model is linked to abnormal aPKC cellular localization. Similarly, given that the Dlg1/GUK complex is required for proper synaptic localization of Scrib in *Drosophila* [47], it follows that Dlg1 mislocalization could trigger the concomitant mislocalization of Scrib. Future studies investigating this hypothesis would be of interest. The mechanism underlying Dlg1 mislocalization remains to be determined. In summary, we showed that in a mouse model of ocular adenocarcinoma, Dlg1, Scrib, and Lgl1 are present in the retina and tumor cells, albeit displaying different patterns of intracellular distribution in comparisons with normal mouse eyes of the same age. These alterations are found in the neural retina and RPE tumor cells as early as at the P15 stage of tumor progression.

We also found a reduction in Dlg1, Scrib, and Lgl1 mRNA and protein levels at the later P30 stage. Consistent with our results, the altered localization of Dlg1, Lgl1, and Scrib appeared to be associated with dysplasic stages whereas their downregulation could be considered as a late stage marker in the progression of ocular tumor development. The downregulation of these proteins and/or changes in their intracellular localization may contribute to the transformation and development of ocular tumors in our mouse model. The mislocalization and downregulation of Dlg1, Scrib, and Lgl1 in our model may alter their normal functions in controlling cell proliferation and cell migration. Further studies are needed to clearly elucidate the molecular and biochemical mechanisms responsible for the different expression patterns and downregulation observed in the RPE adenocarcinoma of our mouse model.

The downregulation of *Dlg1*, *Lgl1* and *Scrib* gene expression observed in Trp1/Tag mice was only detected at P30, a stage corresponding to the beginning of the invasion process. Our results suggest an involvement of these proteins in the dissemination and occurrence of metastases. This could be confirmed by studying the phenotypes of *Drosophila* mutants. Loss of Scribble, Dlg or Lgl leads to the tumorous overgrowth of epithelial tissues in *Drosophila* imaginal discs. The transplantation of these mutated tissues into new *Drosophila* hosts appears to be sufficient to confer migratory properties to the transplanted mutated epithelial cells and result in tumor formation at distant sites in the host [48]. Furthermore, some studies have demonstrated the role of Dlg, Lgl and Scrib in the control of cell migration in *Drosophila*, zebrafish, and mammals [46,49-52]. Consistent with the potential role of these proteins in the migration of tumor cells in our ocular tumor model, we also showed that E-cadherin mRNA is reduced at the P30 and three month stages in our ocular tumoral mouse model. This suggests that Scrib and Lgl1 mouse homologs may regulate cell migration by alterating E-cadherin gene expression. This is consistent with previous findings. In *Drosophila melanogaster*, a *Scrib* mutation in Ras-transformed eye disc cells suppresses E-cadherin expression, inducing invasion of the basement membrane and metastasis [26]. However, the role of Scrib in controlling E-cadherin expression remains unclear. Indeed, unlike in *Drosophila* *melanogaster*, the reduction of Scrib levels in MDCK cells had no effect on E-cadherin expression [52]. In human melanoma, a loss of Hugl1 is associated with E-cadherin downregulation [27]. Although the possibility of E-cadherin regulation by Scrib or Lgl remains undetermined, our results are consistent with a previous study in transgenic mice that strongly suggests that the loss of E-cadherin directly promotes the transition of benign adenoma into carcinoma [53]. We also found that the loss of E-cadherin gene expression was correlated with the upregulation of *N-cadherin* gene expression in our mouse model. Interestingly, the switch from E-cadherin to N-cadherin is a marker of the epithelial to mesenchymal transition (EMT). This abnormal switch leads to the loss of intercellular junctions, provoking the detachment of tumor epithelial clusters. This property is typically displayed by metastasizing cells and is a feature of aggressive tumors [54]. These findings also suggest that Dlg1, Scrib, and Lgl contribute to EMT in our ocular tumor mouse model, a process relevant to human cancers [27]. However, this hypothesis should be confirmed by additional appropriate experiments.

To conclude, this is the first study demonstrating the simultaneous deregulation of expression of the three major suppressor genes conserved between *Drosophila* and mammals. We also demonstrated changes in the behavior of these proteins during ocular carcinogenesis including their mislocalization and downregulation. Both the mislocalization and downregulation of Dlg1, Scrib, and Lgl1 proteins seemed to be correlated to tumor progression, which was determined by the acquisition of new properties in cancer cells, in our mouse model of adenocarcinoma. Our results suggest that alterations of Dlg1, Scrib, and Lgl1 functions could promote tumorigenesis by first promoting hyperplasia and second resulting in a loss of cell polarity, thereby reducing cell adhesion and facilitating aggressive overgrowth and invasive behavior. Studies of biopsies and samples from human ocular adenocarcinomas are now required to confirm the relevance of our findings. Nevertheless, our findings together with previous studies provide increasing evidence that Dlg, Scrib, and Lgl *Drosophil*a homologs are involved in pathways that play a major role in cancer development.
